# The use of bevacizumab in primary malignant tumors of the central nervous system: A single-center experience

**DOI:** 10.1097/MD.0000000000034286

**Published:** 2023-07-28

**Authors:** Metin Pehlivan, Meltem Ekenel

**Affiliations:** a Department of Medical Oncology, Zonguldak Ataturk State Hospital, Zonguldak, Turkey; b Department of Medical Oncology, Istanbul University Institute of Oncology, Istanbul, Turkey

**Keywords:** bevacizumab, central nervous system tumors, oncology, targeted therapy

## Abstract

Primary central nervous system tumors rank 8 among other cancers in patients over 40 years of age. Glioblastoma is the most common primary central nervous system malignancy, accounting for 48 percent of the cases. The present study evaluates the effect of bevacizumab on the disease course in patients who received bevacizumab therapy due to primary central nervous system tumors in our clinic. The study was designed as a retrospective study. The records of patients treated between January 2000 and August 2021 were reviewed and patients who received bevacizumab therapy due to primary central nervous system tumor were included in the study to evaluate the effect of the therapy on disease course among the subgroups of patients. The study included 70 patients. Of these patients, 40 were male (57.1%) and 30 (42.9%) were female. The median duration of follow-up was 28 months (8–209 months). The median age of the patients was 47 years. The median duration of exposure to bevacizumab was 5 months (1–33 months). Forty-nine patients (71.4%) were histologically diagnosed with glioblastoma multiforme. The median progression-free survival (PFS) was 5 months (95% confidence interval 4–6). The median overall survival (OS) was 8 months (95% confidence interval 6.97–9.02). No statistically significant difference in OS or PFS was observed in any patient subgroup. The therapy was discontinued only in 2 patients (2.9%) due to side effects (1 patient with pulmonary embolism and 1 patient with intracranial hemorrhage). The present study found that the use of bevacizumab is safe in terms of side effects. No statistically significant difference in OS or PFS was observed in any patient subgroup. There is a need for studies on a larger number of patients to find out which patient subgroup benefit the most from bevacizumab therapy.

## 1. Introduction

According to the World Health Organization’s 2021 classification, central nervous system tumors are a heterogeneous group of diseases with various histological subtypes.^[1]^ Primary central nervous system tumors rank 8 among other cancers in patients over 40 years of age.^[2]^ The incidence of primary malignant tumors of the central nervous system is 2.1 to 5.8 cases per 100,000 people.^[3]^ Glioblastoma is the most common primary central nervous system malignancy, accounting for 48% of the cases.^[4]^ Glioblastomas are malignancies with poor prognosis with a median overall survival (OS) of 15 months.^[5]^ The basis of therapy in primary central nervous system tumors is surgery, but radiotherapy and chemotherapy are also used. Bevacizumab is a drug that is often used in patients with relapsing primary central nervous system tumors. In the present study, patients with primary central nervous system tumors receiving bevacizumab therapy in our clinic were divided into subgroups according to various parameters and the effect of bevacizumab therapy on the disease course was evaluated. The aim of our study is to compare the clinical course of bevacizumab in different patient subgroups and to examine in which patient subgroup bevacizumab may be more beneficial.

## 2. Materials and method

### 2.1. Study design and patient characteristics

The study was designed as a retrospective study. The study included patients aged >18 years who received at least 1 dose of bevacizumab due to primary central nervous system tumor in the Medical Oncology Clinic of İstanbul University Oncology Institute. Bevacizumab was administered every 14 days at a dose of 10 mg/kg. The records of patients treated between January 2000 and August 2021 were reviewed and patients who received bevacizumab therapy due to primary central nervous system tumor were included in the study.

### 2.2. Clinical parameters studied

Patients’ age, gender, histological tumor subgroups, history of surgery, adjuvant therapies received, number of relapses after which bevacizumab was administered, chemotherapy agents administered together with bevacizumab, reason for discontinuation of bevacizumab therapy, progression-free survival (PFS) benefit of bevacizumab (calculated as the time from the initiation of bevacizumab therapy to disease progression), OS benefit of bevacizumab (calculated as the time from the initiation of bevacizumab therapy to all-case death), and final status of the patients were examined.

### 2.3. Statistical analysis

The R-Studio software package was used in statistical analysis. Qualitative variables were expressed as frequency and percentage, and quantitative variables were expressed as median with minimum and maximum values. The Kaplan–Meier method was used to evaluate survival, and OS and PFS were presented within 95% confidence interval (CI). The factors affecting OS and PFS were analyzed using the log-rank test. A *P* value of <.05 was considered statistically significant in all statistical analyses.

### 2.4. Ethics committee approval

The academic committee approval was obtained from the Academic Board of İstanbul University Oncology Institute and the ethics committee approval was obtained from the Ethics Committee of İstanbul University İstanbul Faculty of Medicine. The researchers have read the latest version of the World Medical Association Declaration of Helsinki and the Good Clinical Practices Guidelines/Good Laboratory Practices Guidelines published recently by the Ministry of Health and conducted the research accordingly.

## 3. Results

**Figure 1.**
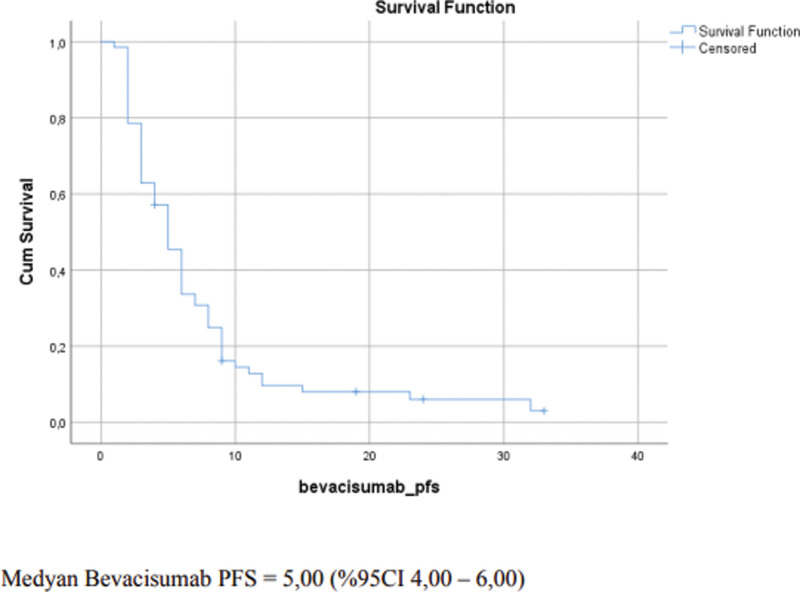
The median progression-free survival (PFS) in patients receiving bevacizumab therapy.

The study included 70 patients. Of these patients, 40 were male (57.1%) and 30 (42.9%) were female. The median duration of follow-up was 28 months (8–209 months). The median age of the patients was 47 years. Histologically, 49 patients (70%) were diagnosed with glioblastome multiforme (GBM), 6 patients (8.6%) were diagnosed with anaplastic diffuse glial tumor, 5 patients (7.1%) were diagnosed with grade 3 oligodendroglioma, 3 patients (4.3%) were diagnosed with diffuse astrocytoma, 3 patients (4.3%) were diagnosed with anaplastic oligodendroglioma, 3 patients (4.3%) were diagnosed with grade 2 oligodendroglioma, and 1 patient was diagnosed with small-cell glioblastoma. Sixty-seven patients (95.7%) underwent surgery and 3 patients (4.3%) were not operated on. Fifty-nine patients received adjuvant chemotherapy, 10 patients received radiotherapy alone, and 1 patient received no adjuvant therapy. The median time to relapse was 13 months (4–107 months). Of the relapsing patients, 20 (28.4%) underwent surgery (gross total excision in 18 and subtotal excision in 2 patients) and 50 patients were not operated on (71.2%). Bevacizumab was initiated at the time of diagnosis in 1 patient (1.4%), after the first relapse in 52 patients (74.3%), after the second relapse in 7 patients (10%), after the third relapse in 8 patients (11.4%), and after the fourth relapse in 2 patients (2.9%). In combination with bevacizumab therapy, 54 patients (77.1%) received irinotecan, 1 patient (1.4%) received temozolomide, 1 patient (1.4%) received capecitabine, and 14 patients (20%) received no chemotherapeutic agent (Table 1). The median duration of exposure to bevacizumab was 5 months (1–33 months). The median PFS in patients receiving bevacizumab therapy was 5 months (95% CI 4–6) (Fig. 1). Of the patients 8.6% were progression-free at 12 months, 2.9% were progression-free at 24 months, and no patient without disease progression remained at the end of 60 months. The median PFS was 4 months in female patients and 6 months in male patients. The difference was not statistically significant (*P* = .143). The median PFS was 4 months (1–33 months) in patients who were not operated on after disease relapse and 6 months (2–32 months) in those who underwent surgery after disease relapse. The difference was not statistically significant (*P* = .339). The median PFS was 5 months in patients who were placed on bevacizumab therapy at the time of initial diagnosis or after the first relapse and 5 months in those who received bevacizumab therapy after the second and subsequent relapses (Table 2). The difference was not statistically significant (*P* = .639). In the analysis of PFS among the pathological subgroups, the median PFS was 4 months in patients diagnosed with GBM and 5 months in those with other diagnoses. The difference was not statistically significant (*P* = .884). The median PFS was 4 months (95% CI 2.03–5.97) in patients who received bevacizumab therapy within 12 months after the diagnosis, 6 months (95% CI 5.02–6.98) in patients who received bevacizumab therapy within 13–23 months after the diagnosis, and 5 months (95% CI 2.13–7.87) in those who received bevacizumab therapy at 24 months or after. The difference was not statistically significant (*P* = .809).

**Table 1:** Gender distribution, histological tumor subgroups, number of patients who underwent surgery, number of patients who received which adjuvant therapies, number of patients who underwent surgery after relapse, procedures performed in patients undergoing surgery, number of relapses after which bevacizumab therapy was initiated, receipt of chemotherapy in combination with bevacizumab therapy, chemotherapy regimens received in combination with bevacizumab therapy, reasons for the discontinuation of bevacizumab therapy, and final status of the patients

	n	%
Gender		
Male	40	57.1
Female	30	42.9
Pathology		
Diffuse astrocytoma	3	4.3
Oligodendroglioma grade 3	5	7.1
Anaplastic diffuse glial tumor	6	8.6
Anaplastic oligodendroglioma	5	4.3
Glioblastome multiforme	49	70.0
Oligodendroglioma grade 2	3	4.3
Small-cell glioblastoma	1	1.4
Operated on		
Yes	67	95.7
No	3	4.3
Adjuvant therapy		
Not received	1	1.4
Radiotherapy	10	14.2
Chemoradiotherapy	59	85.5
Operation after relapse		
Yes	20	29,0
No	50	71.4
Procedure after relapse		
Gross total excision	18	90.0
Subtotal excision	2	10.0
Number of relapse after which bevacizumab therapy was initiated		
At the time of diagnosis	1	1.4
1	52	74.3
2	7	10.0
3	8	11.4
4	2	2.9
Chemotherapy combined with bevacizumab therapy		
Not received	14	20.0
Irinotecan	54	77.1
Temozolomide	1	1.4
Capecitabine	1	1.4
Discontinuation of bevacizumab therapy		
Progression	50	71.4
Treatment-free follow-up after achieving treatment response	2	1.5
Pulmonary embolism	1	1.5
Deterioration in general condition	14	20
Patients’ preferences	2	3.0
Intracranial hemorrhage	1	1.5
Final status		
Died	65	92.9
Survived	5	7.1

**Table 2:** OS and PFS according to the number of relapses after which bevacizumab therapy was initiated

	Newly diagnosed-first relapse	Second and subsequent relapses	*P*
Bevacizumab OS	8 (2–66)	8 (3–64)	.685
Bevacizumab PFS	5 (1–33)	5 (2–32)	.639

**Figure 2.**
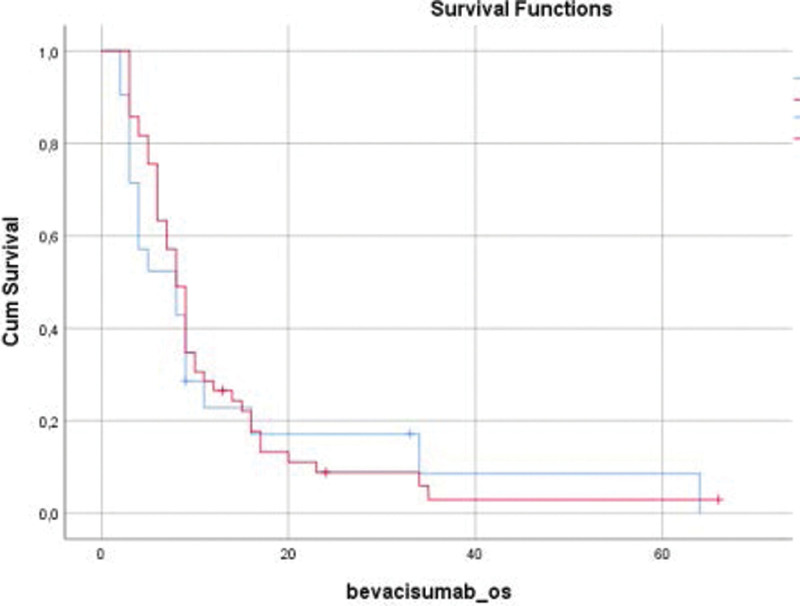
Kaplan–Meier curve for overall survival (OS) with bevacizumab therapy according to the histological subgroups.

The OS in patients receiving bevacizumab therapy was 8 months (95% CI 6.97–9.02). The OS was 24.3% at 12 months, 2.9% at 24 months and 2.9% at 60 months. The median OS was 8 months in female patients and 7 months in male patients. The difference was not statistically significant (*P* = .863). The median OS was 8 months in patients who were not operated on after disease relapse and 8.5 months in those who underwent surgery after disease relapse. The difference was not statistically significant (*P* = .067). The median OS was 8 months in patients who were placed on bevacizumab therapy at the time of initial diagnosis or after the first relapse and 8 months in those who received bevacizumab therapy after the second and subsequent relapses. The difference was not statistically significant (*P* = .685). In the analysis of OS among the pathological subgroups, the median OS was 8 months in patients diagnosed with GBM and 8 months in those with other diagnoses. The difference was not statistically significant (*P* = .750, Fig. 2). The median OS was 9 months (95% CI 7.31–10.69) in patients who received bevacizumab therapy within 12 months after the diagnosis, 8 months (95% CI 6.62–9.38) in patients who received bevacizumab therapy within 13–23 months after the diagnosis, and 6 months (95% CI 1.20–10.80) in those who received bevacizumab therapy at 24 months or after. The difference was not statistically significant (*P* = .341).

Eighteen patients (25.7%) received therapy with a chemotherapeutic agent after discontinuation of bevacizumab for some reason, while 52 patients (74.3%) received no therapy. The median PFS was 5 months in patients who were started on another therapy after discontinuation of bevacizumab therapy for some reason and 5 months in those who did not receive any form of therapy. The difference was not statistically significant (*P* = .642). The median OS was 7 months in patients who were started on another therapy after discontinuation of bevacizumab therapy for some reason and 9 months in those who did not receive any form of therapy. The difference was not statistically significant (*P* = .252).

Treatment-free follow-up was performed in 50 patients (71.4%) who showed disease progression, in 14 patients (20%) due to deterioration in general condition, in 2 patients (2.9%) based on the patients’ preferences, in 2 patients (2.9%) due to development of side effects (1 patient had pulmonary embolism and 1 patient had intracranial hemorrhage), and in 2 patients (2.9%) due to achievement of maximum response. In the analysis of the final status of the patients, 65 patients (92.8%) died and 5 patients (7.2%) survived.

## 4. Discussion

There is no consensually agreed therapy for the treatment of relapsing primary central nervous system tumors. Surgery and radiotherapy are options but systemic agents are also available. The present study evaluated patients receiving bevacizumab therapy.

In a phase 2 BELOB study^[6]^ evaluating 153 patients receiving bevacizumab alone, lomustine alone or bevacizumab in combination with lomustine, the median PFS was 3 months (3–4 months) in the bevacizumab alone arm, 1 month (1–3 months) in the lomustine alone arm, and 4 months (3–8 months) in the combined bevacizumab and lomustine arm. The median OS was 8 months (6–9 months) in the bevacizumab alone arm, 8 months (6–11 months) in the lomustine alone arm, and 12 months (8–13 months) in the combined bevacizumab and lomustine arm. In the present study, the median PFS was 5 months and the median OS was 8 months. Numerically better median PFS in the present study may be caused by higher median age of the patient population (median age was 58 years in the bevacizumab alone arm, 56 years in the lomustine alone arm, and 58 years in the combined bevacizumab and lomustine arm) and inclusion of only patients with a histological diagnosis of glioblastoma in the BELOB 2 study.

In a study by Henry Friedman et al comparing bevacizumab as a single agent with bevacizumab and irinotecan combination in 167 patients,^[7]^ the median PFS was 4.2 months in the bevacizumab alone arm and 5.6 months in the combined bevacizumab and irinotecan arm. The median OS was 9.2 months in the bevacizumab alone arm and 8.7 months in the combined bevacizumab and irinotecan arm. In the present study, 77% of the patients received bevacizumab therapy in combination with irinotecan and similar OS and PFS values have been achieved. The therapy was discontinued due to side effects in only 2.9% of the patients in the present study, while 17.7% of the patients in the combined bevacizumab and irinotecan arm required discontinuation therapy in the study by Friedman et al. Our study is different in this regard.

In a study comparing lomustine alone with the combination of lomustine and bevacizumab in 437 patients,^[8]^ the median PFS was 1.5 months in the lomustine alone arm and 4.2 months in the lomustine + bevacizumab arm. The median OS was 8.6 months in the lomustine alone arm and 9.1 months in the lomustine + bevacizumab arm. When compared to our study, median PFS and OS values in their study were similar to those found in the present study. In terms of side effects, pulmonary embolism was encountered in approximately 5% of the patients in their study. Pulmonary embolism was observed in only 1 patient (1.4%) in the present study.

In a study examining the use of bevacizumab as a single agent in 22 patients with oligodendroglioma,^[9]^ the median PFS was 6.75 months and the median OS was 8.5 months. In the present study, only 3 patients had a histological diagnosis of oligodendroglioma with similar OS but lower PFS. Lower PFS in the present study can be explained by higher number of patients with a histological diagnosis of glioblastoma multiforme.

In a study examining the use of bevacizumab as a single agent in 25 patients with anaplastic astrocytoma,^[10]^ the median PFS was 6 months and the median OS was 9 months. PFS at 12 months was found to be 20 percent. The median in their study was 50 years similar to the median age in the present study. Median PFS and OS were similar to those in the present study; however, PFS at 12 months was higher in their study. This difference can be explained by the predominance of patients with a histological diagnosis of glioblastoma multiforme.

In the present study, no statistically significant difference in OS or PFS was observed in any patient subgroup. The authors of the present study consider that this was a considerably important finding. One question still awaits a response: “Which patients are eligible for bevacizumab therapy?”. Compared to other studies, it is noted that OS and PFS were similar but side effects interfering with the treatment were quite lower in the present study. This is the most remarkable finding differentiating the present study from the others.

## 5. Limitations

Important limitations of the present study include retrospective study design, small number of patients, not having determined the histological subtypes in some patients, and not having studied mutations such as O-6-methylguanine-DNA methyltransferase, Alpha-thalassemia/mental retardation syndrome X, and isocitrate dehydrogenase-1.

## 6. Conclusion

Primary central nervous system tumors constitute a group of malignancies that have a poor clinical course and limited treatment options. The present study found that the use of bevacizumab is safe in terms of side effects. There is a need for studies on a larger number of patients to find out which patient subgroup benefit the most from bevacizumab therapy.

## Author contributions

**Conceptualization:** Metin Pehlivan.

**Data curation:** Metin Pehlivan.

**Formal analysis:** Metin Pehlivan.

**Investigation:** Metin Pehlivan.

**Methodology:** Metin Pehlivan.

**Supervision:** Meltem Ekenel.

**Writing – original draft:** Metin Pehlivan.
